# Single-Cell Pulsed-Field Gel Electrophoresis to Detect the Early Stage of DNA Fragmentation in Human Sperm Nuclei

**DOI:** 10.1371/journal.pone.0042257

**Published:** 2012-07-27

**Authors:** Satoru Kaneko, Joji Yoshida, Hiromichi Ishikawa, Kiyoshi Takamatsu

**Affiliations:** 1 Reproduction Center, Gynecology, Ichikawa General Hospital, Tokyo Dental College, Ichikawa, Chiba, Japan; 2 Reproduction Center, Urology, Ichikawa General Hospital, Tokyo Dental College, Ichikawa, Chiba, Japan; Universita’ di Milano, Italy

## Abstract

Single-cell pulsed-field gel electrophoresis (SCPFGE) with dual electrode pairs was developed to detect the early stage of DNA fragmentation in human sperm. The motile sperm were purified by the commonly used density-gradient centrifugation technique and subsequent swim-up. The sperm were embedded in a thin film of agarose containing bovine trypsin (20 µg/mL) and were then lysed. Prior to SCPFGE, proteolysis of DNA-binding components, such as protamine and the nuclear matrix was essential to separate the long chain fibers from the fibrous and granular fragments derived from a single nucleus. The overall electrophoretic profiles elucidated the course of DNA fragmentation. A few large fibrous fragments were observed at the beginning of the process, however, as the fragmentation advanced, the long chain fibers decreased and shortened, and, conversely, the granular fragments increased until finally almost all the DNA was shredded. Although the ejaculate contained sperm with heterogeneous stages, the purified motile sperm exhibited several dozens of uniformly elongated fibers arising from the tangled DNA at the origin, whereas a part of these fibers gave rise to fibrous fragments beyond the tip of the elongated fibers, and their numbers and sizes varied among the sperm. Conventional intra-cytoplasmic sperm injection (ICSI) usually depends on intra-operative light microscopic observation to select a sperm for injection. The present results revealed that sperm motility could not give full assurance of DNA integrity. SCPFGE is likely to serve an important role in the preoperative differential diagnosis to determine the competence of the sperm population provided for injection.

## Introduction

It is well known that human ejaculate contains a heterogeneous sperm population that possesses a variety of abnormalities. In assisted reproductive technology (ART) in the clinical setting, the nuclear deterioration of human sperm, in particular, DNA fragmentation as a consequence of double-strand breaks, attracts attention. Various types of DNA damage have been studied; of these, the most problematic are probably double-strand breaks because repair of such lesions is intrinsically more difficult as compared to other lesions [1]. Although cells can adapt to low levels of irreparable damage, as little as one double-strand break in DNA can be sufficient to kill a cell if it inactivates an essential gene or triggers apoptosis [2]. Moreover, mature sperm have few DNA repair mechanisms [3], and the capacity and accuracy of human embryonic DNA break repair in culture environment are still remain unclear [4], [5]. If a sperm with damaged DNA is incorporated into the embryonic genome, it may lead to sperm-derived chromosomal aberrations [5], which may in turn result in higher miscarriage rates [6] and an increased risk of pregnancy loss [7]. The resultant aberrations can also be potentially inherited through the germ line for generations [8]–[10]. Several studies have reported that the rate of DNA damage in sperm increases in infertile men with poor semen quality, who are the primary subjects for intra-cytoplasmic sperm injection (ICSI) [6], [11]. Although the techniques for sperm injection in clinical ICSI are well established, the sperm is selected merely based on motility and gross morphology, as observed under a microscope, and there are no validated methods to address and assure sperm nuclear DNA integrity.

To date, several methods based on different principles have been proposed to observe DNA cleavages in human sperm nuclei. The sperm chromatin dispersion test [12] is based on the observation that sperm with fragmented DNA fail to produce the characteristic halos of dispersed DNA loops as observed in sperm with intact DNA. The terminal deoxynucleotidyl transferase-mediated dUTP nick-end labeling (TUNEL) assay [13], [14] has been employed to estimate the amount of double-strand breaks based on the intensity of incorporated fluorescent dUTP. The comet assay [15], [16] is used to separate DNA fragments by electrophoresis, and the amounts of both single- and double-strand DNA breaks are measured using an alkali pH method [17].

Although there is some scope for DNA break repair after penetration into the oocyte, the permissible limit of DNA fragmentation in a sperm, which enables proper embryogenesis and subsequent fetal development, may be null or extremely low [2]. Moreover, from the view of clinical ART, it is essential to detect the early stages of DNA fragmentation. Hence, in the present study, we newly developed single-cell pulsed-field gel electrophoresis (SCPFGE) to quantitatively determine the number and size of DNA fragments derived from a single sperm nucleus.

**Figure 1:**
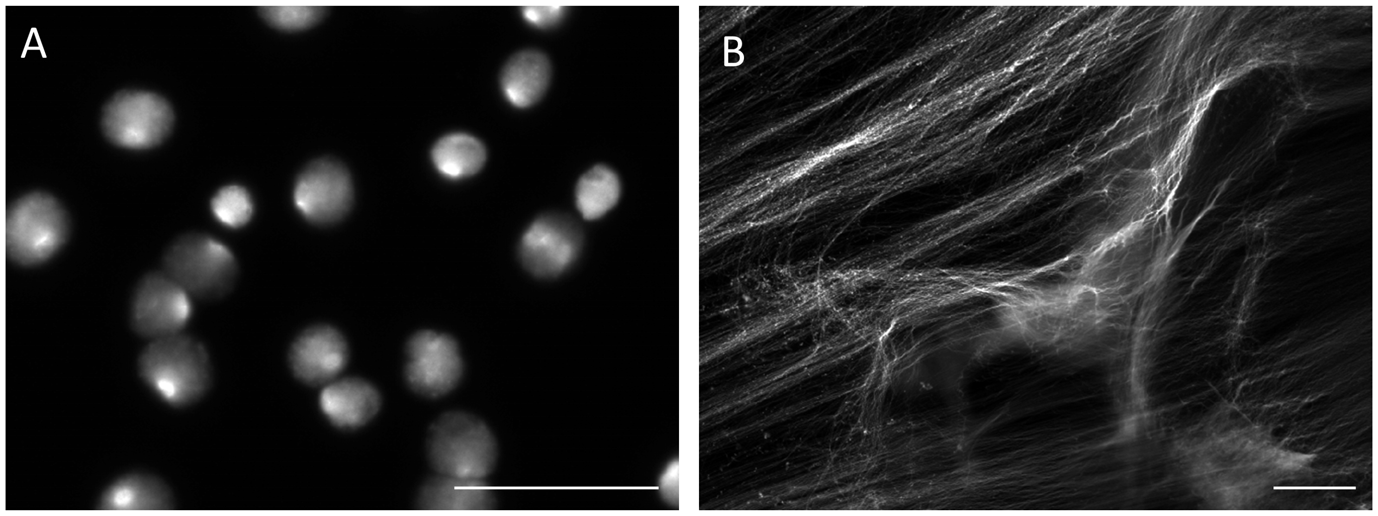
Significance of trypsin proteolysis in dissociating DNA from intracellular DNA-binding components. A: The purified sperm fraction was concentrated to give 2×10^7^ cells/ml, then equal volumes of the resulting suspension and 2 times concentrated the cell lytic reagents containing Cyber-Gold (2×10^4^ diluted) were mixed, B: Trypsin (20 µg/mL) was subsequently added to the above mentioned specimen. An aliquot (10 µL) was put on a glass slide and mounted with a cover slip.

**Figure 2:**
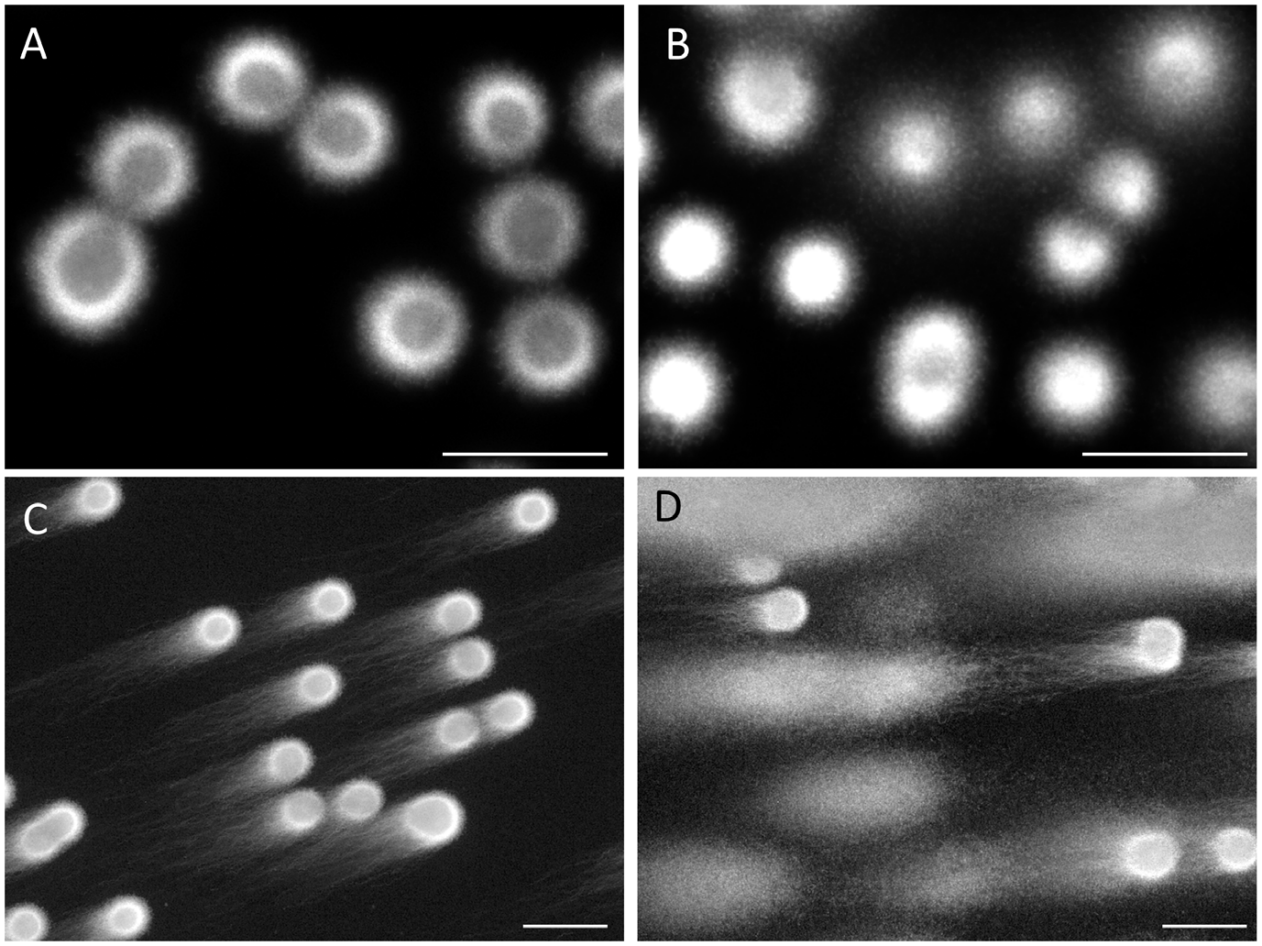
Comparison of the embedded sperm that were lysed with trypsin between the purified fraction and ejaculate. After treatment with the cell lytic agent; A: the purified sperm, B: the ejaculate. Electrophoretic profiles after SCPFGE; C: the purified sperm, and D: the ejaculate.

## Materials and Methods

### Preparation of Human Sperm with Progressive Motility

**Figure 3:**
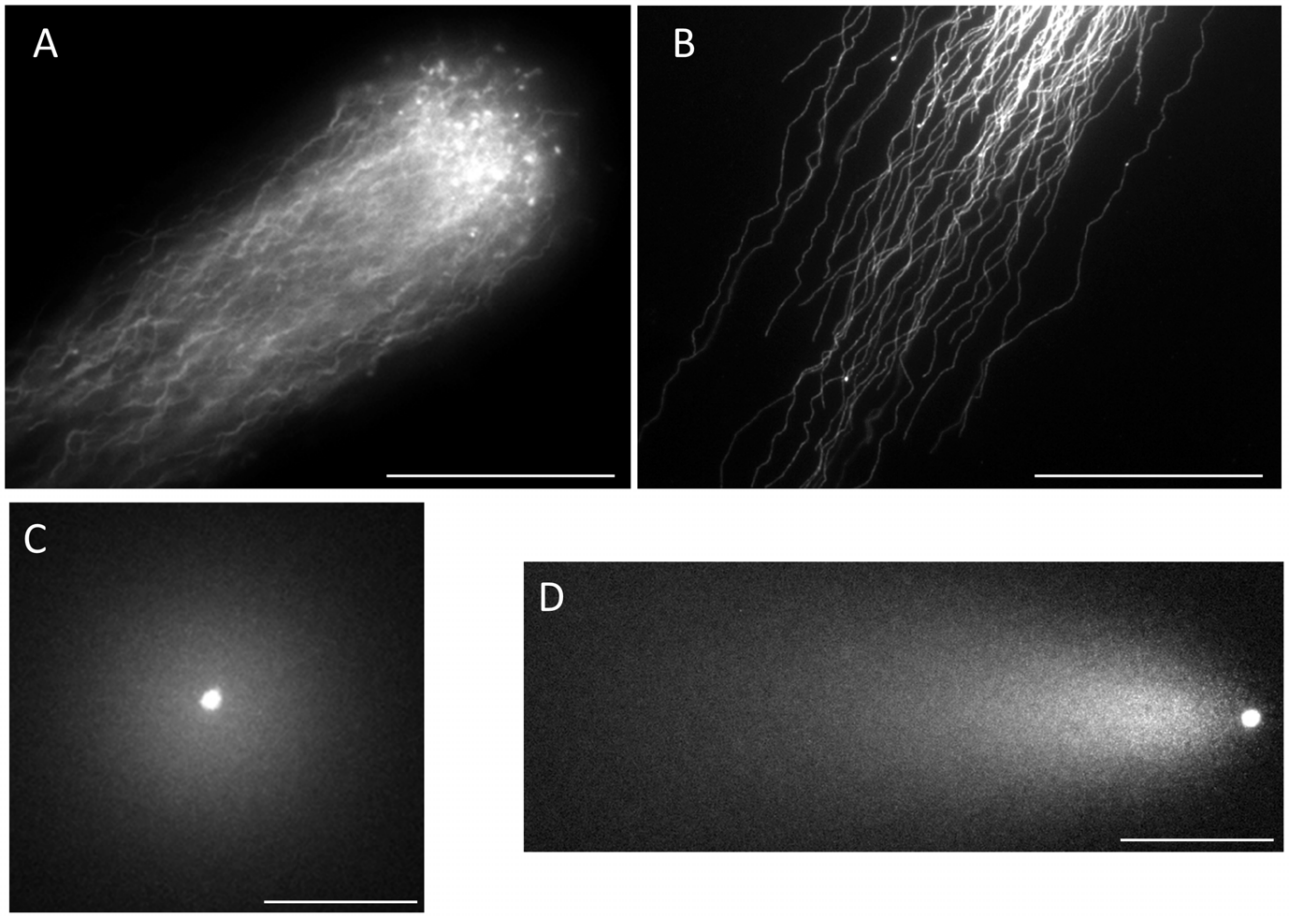
Typical profiles of intact DNA fibers and of DNA at the end stage of fragmentation. The elongated long chain fibers; A: near the origin and B: apical region, C: granular fragments dispersed around after the lysis, and D: granular fragments fanned out after electrophoresis.

**Figure 4:**
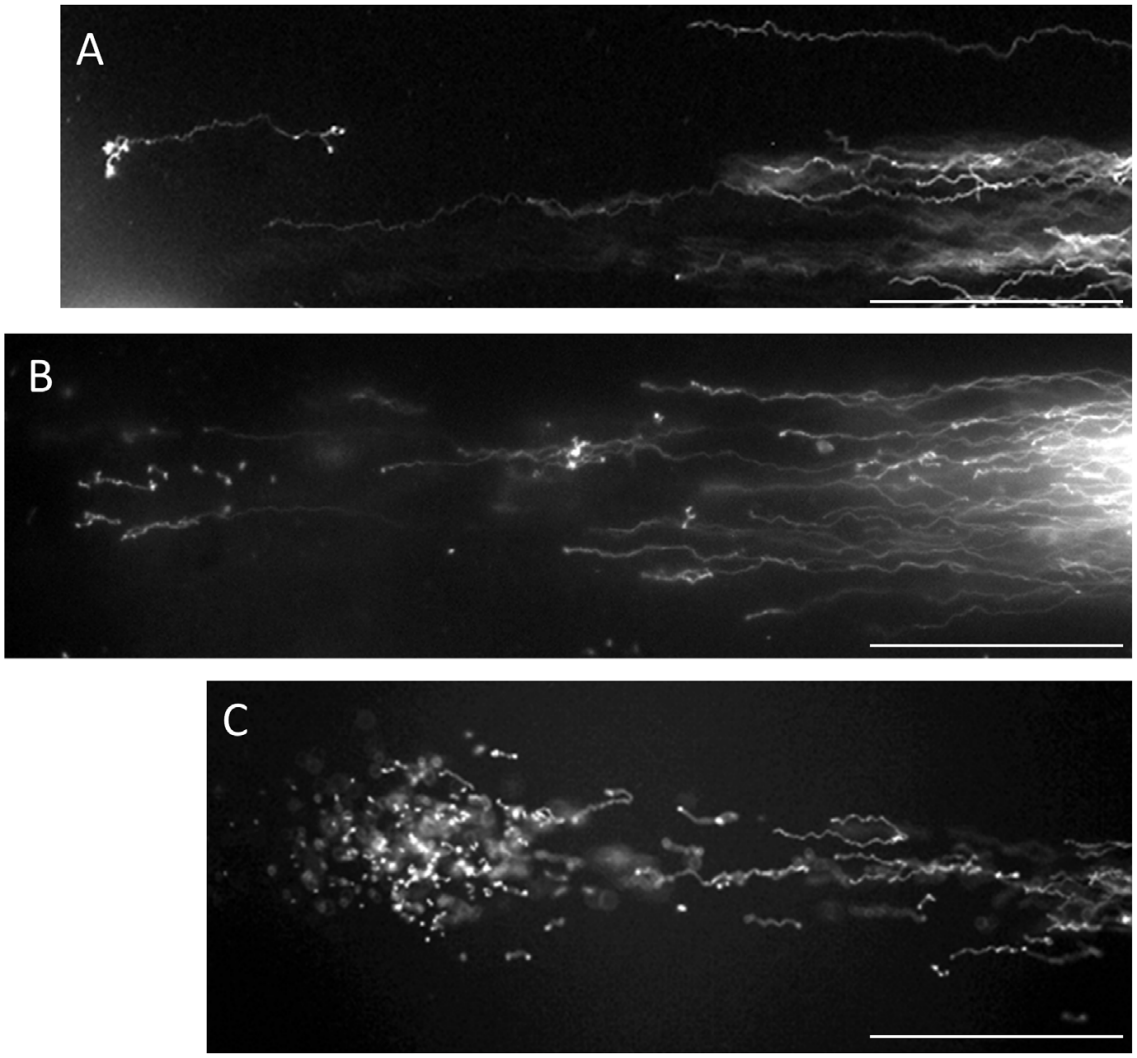
Fibrous fragments separated beyond the anterior end of the elongated fibers.

**Figure 5:**
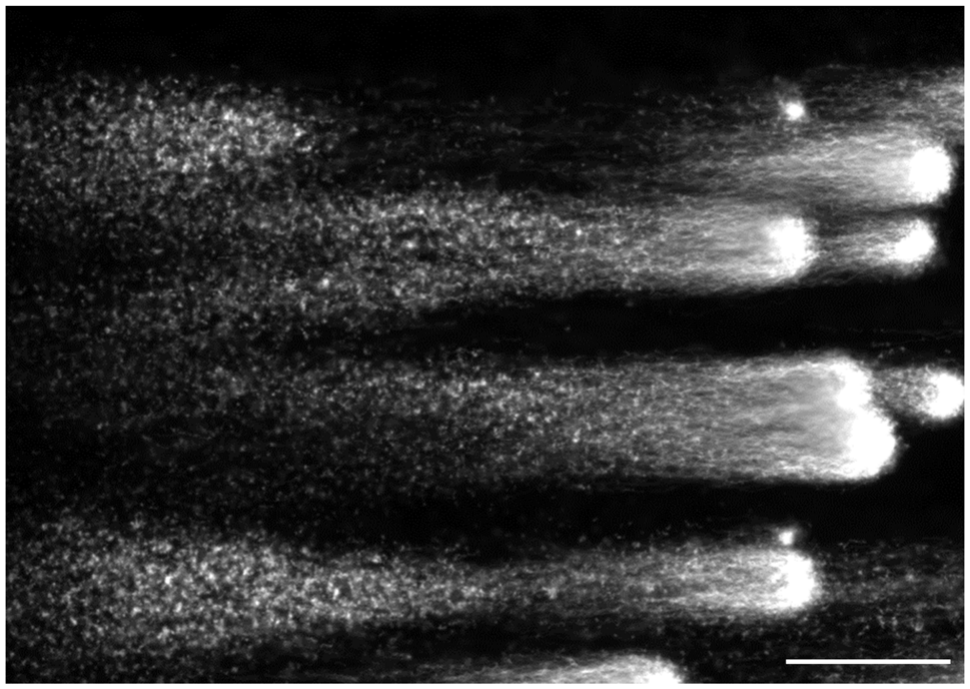
Effect of deoxyribonuclease I on the electrophoretic profile of the long chain DNA fibers.

**Figure 6:**
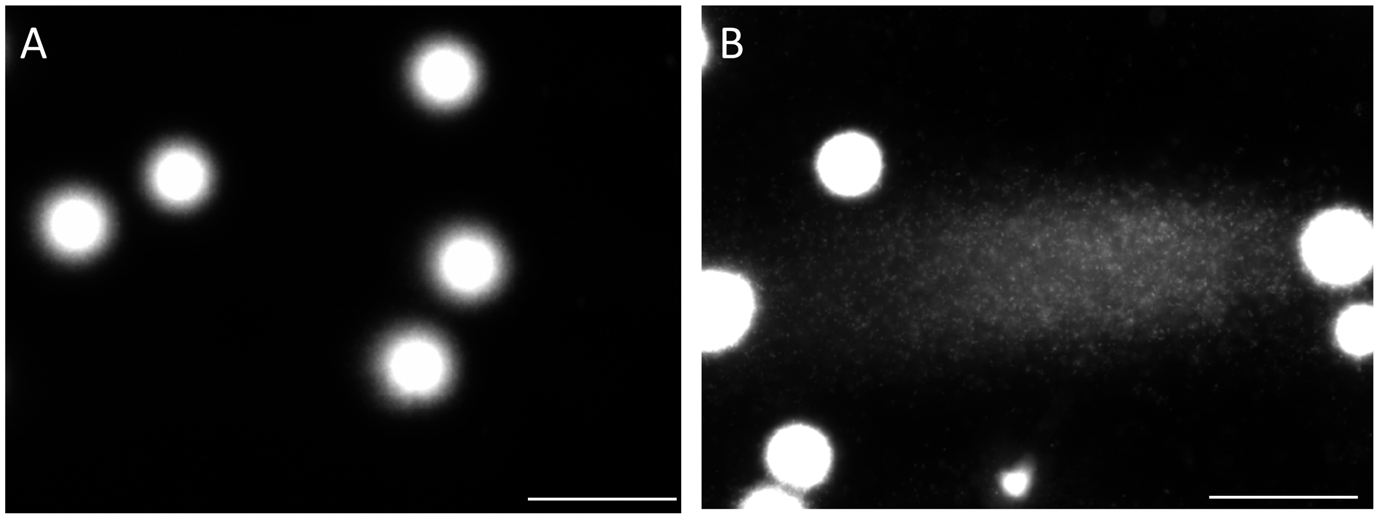
Comparison of the embedded sperm that were lysed without trypsin between the purified fraction and the ejaculate. Electrophoretic profiles after SCPFGE; A: the purified sperm, and B: the ejaculate.

**Figure 7:**
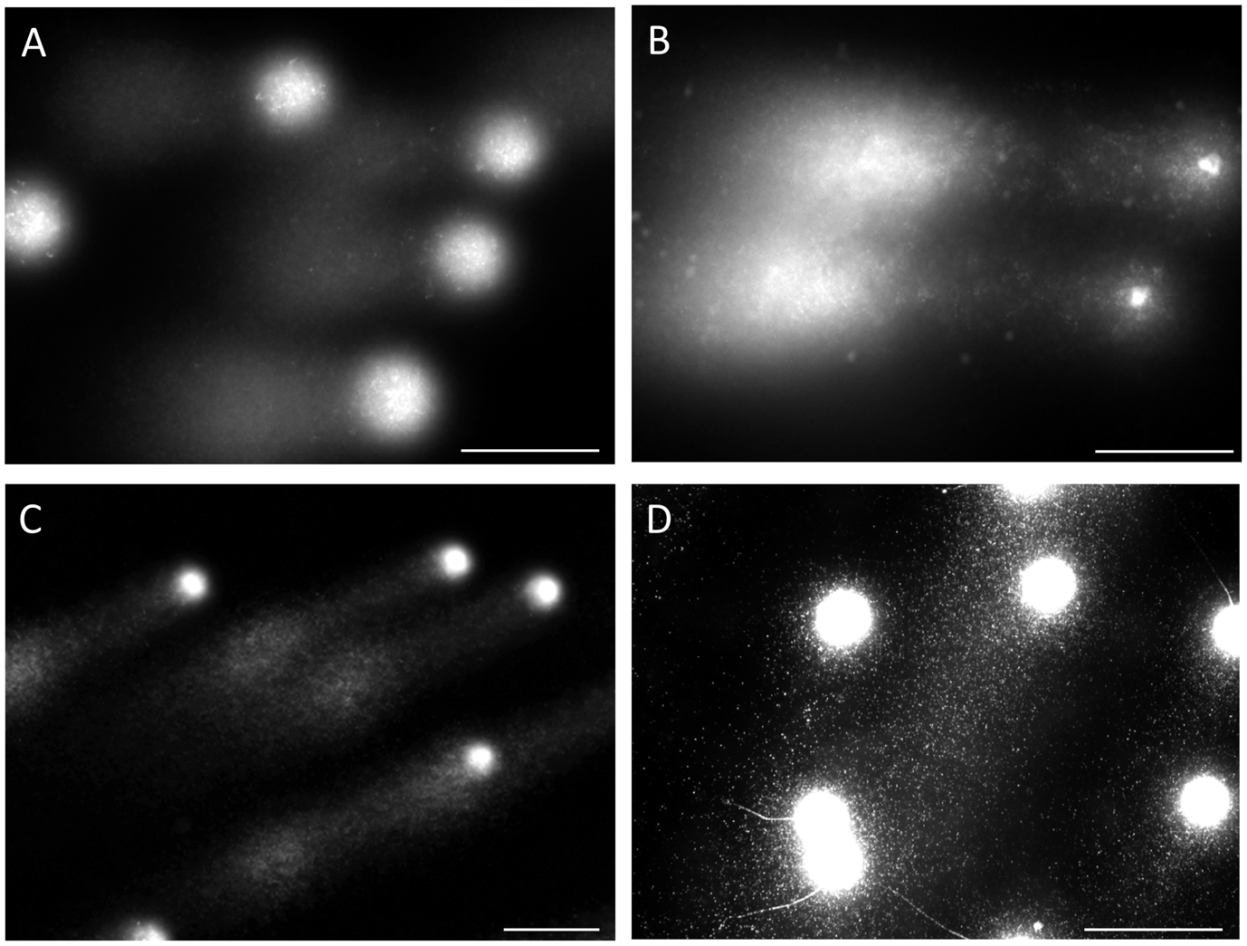
Effects of alkali on the purified sperm that were lysed with or without trypsin. The sperm lysed with trypsin were treated with A: 0.05 or B: 0.1 mol/L NaOH prior to SCPFGE, C: treated with 0.1 mol/L NaOH after electrophoresis, and D: the sperm lysed without trypsin were treated with 0.1 mol/L NaOH prior to SCPFGE.

Ejaculates were obtained from volunteers or patients who visited our clinic, all the study participants who provided the ejaculates received explanations on the aim of this study, the procedures of semen processing and the measurement items, then gave the consent in writing the form. The ethical committee of Ichikawa General Hospital specifically approved this study. Sperm concentration and motility were measured with a computer-assisted image analyzer (C-Men, Compix Inc., PA, USA). Sperm with progressive motility were prepared as follows: the ejaculate was diluted twice with 20 mM HEPES-buffered Hank’s solution and 2.0 mg/mL human serum albumin at a pH of 7.4. Next, a variety of particles such as fine urethral calculus, mucinous gel, and fibers were removed by gravity sedimentation and by subsequent filtration with an ART filter (20 µm mesh clearance, Nipro, Japan). Five milliliters of 20 mM HEPES-buffered 98% Percoll (GE Healthcare, NJ, USA) and 2.0 mg/mL human serum albumin at a pH of 7.4 was made isotonic with powdered Hank’s mixture, and were placed in a conical-tip test tube. One milliliter of Hank’s solution was layered on the isotonic Percoll. The tube was set in a tube holder at an angle of 30° and followed by rotation of 10 revolutions (1 revolution/sec) to make density gradient around the interface between Percoll and Hank’s solution. The prepared sperm suspension was centrifuged in a swing-out rotor at 400×*g* for 30 min [18], [19]. Thereafter, the sperm with progressive motility was separated using the swim-up method. Briefly, Hank’s solution (2.0 mL) was overlaid onto the precipitate (200 µl) and allowed to stand for 30 min at 37°C. The motile sperm that swam up into the supernatant was collected. The results shown in Figs. 1, 2, 3, 4, 5, 6 and 7 were obtained by using the sperm prepared as follows: the ejaculate (vol. = 3.6 mL, conc. = 44×10^6^ sperm/mL, motility = 37%), the precipitate of the density-gradient (200 µL, 220×10^6^ sperm/mL, 84% motility) and the swim-up fraction (1.0 mL, 6×10^6^ sperm/mL, 98% motility).

### Single-cell Pulsed-field Gel Electrophoresis (SCPFGE)

An aliquot of human sperm (2×10^4^ cells) was applied to a 7×7 mm area of amino propyl-silane-conjugated glass slides by means of centrifugal auto-smear (Cyto-Tek, SAKURA, Tokyo, Japan). Commercially available twice-crystallized bovine pancreatic trypsin was further purified by affinity chromatography using lima bean trypsin inhibitor (LBTI)-conjugated Sephacryl to remove auto-digested trypsin and some impurities such as deoxyribonucleases (DNases). The adsorbent was pre-equilibrated with 20 mM Tris-HCl (pH 8.3), 1.0 mM CaCl_2_, and trypsin dissolved in the same buffer, and was applied to the column. Unadsorbed materials were washed out with the same buffer containing 0.2 M NaCl until the absorbance value at 280 nm dropped below 0.01. The trypsin-LBTI complex was finally dissociated and eluted from the column using 2.0% acetic acid (pH 1.8). The preparation was diluted with 2.0% acetic acid to yield a trypsin concentration of 200 µg/mL and cryopreserved until use.

The melted 0.56% agarose (0.1 M sodium acetate (pH 4.7) with 0.05% Triton X-100) was filtered with a 0.22 µm pore membrane, and then an aliquot (540 µl) was mixed with 60 µl of the purified trypsin (200 µg/mL) at 40°C. The sperm-containing area of the glass slide was embedded in agarose (2.5 cm×2.5 cm, 50 µm thickness) and chilled for 30 min. The gel was incubated in the cell-lysis reagent (30 mM Tris-polyphosphoric acid, 8.2 mM sodium hexa-metaphosphate, 0.05% Triton X-100, 5.0 mM dithiothreitol, at pH 8.1) at 37°C for 30 min. Prior to the cell lysis, the sperm was incubated with bovine pancreatic deoxyribonuclease I (DNase I, 1.0 U/ml, 50 mM Tris-HCl, pH 8.0, 0.5 mM MnCl_2_, 0.05% Triton X-100) at 37°C for 30 min to observe the influence of endonuclease.

**Table 1:** Number of DNA fragments in the purified sperm with progressive motility.

	Motility (%)		Number of Fragments (%)		
	Ejaculate	Swim up	0	1–10	>11
1	72	97	87.2	3.42	9.40
2	38	94	83.8	6.57	9.60
3	64	96	83.6	3.74	12.6
4	40	91	83.2	12.8	4.00
5	52	92	80.2	13.2	6.58
6	48	95	75.6	12.0	12.4
7	82	93	69.3	19.3	11.4
8	76	96	58.9	22.3	18.8
Mean ± SD	59±17	94±2.1	77.7±9.47	11.7±6.88	10.6±4.44

The sample images were shown in Fig. 4-A and -B were categorized as “1–10 fragments”, and those in Fig. 4-C as “more than 11 fragments”.

The apparatus for SCPFGE was composed as follows: the gel film on the glass slide was placed horizontally at the center of the lower flatbed chamber (20 cm×20 cm). The upper chamber (19 cm×19 cm), which had dual electrode pairs placed in an orthogonal array, was mounted to leave a 5.0 mm gap between the two chambers. The electrode solution and the running buffer (30 mM Tris-polyphosphoric acid at pH 8.1) were connected through a thin slit (0.2 cm×15 cm) filled with 1.0% agarose at the bottom panel of the mounted chamber to minimize the diffusion of reactive oxygen species generated at the electrodes. SPCFGE was performed at 1.5 V/cm with 3.0 sec intervals for 7 min. To observe the effect of alkali on DNA fibers, the lysed sperm were treated with 0.05 mol/L or 0.1 mol/L NaOH for 10 min at ambient temperature. DNA in the gel was stained with diluted (×10^4^) Cyber-Gold (Molecular Probes, Oregon, USA) and was observed under an epifluorescent microscope (excitation: 495 nm, emission: 537 nm). The bar at lower right of the figures expressed 100 µm.

The present study classified the electrophoretic profiles of DNA according to their sizes, and named to be granular fragment, fibrous fragment, and long chain fiber, respectively. The granular fragment was observed as a small particle, the long chain fiber was elongated from the origin without interruption, and the fibrous fragment was recognized as a fiber which was separated beyond the anterior end of the elongated long chain fibers. The number of DNA fragments in a single sperm was counted by microscopic visual observation, more than 200 sperm were observed in each specimen. Values were tentatively classified into three categories: no fragments, 1–10 fragments, and more than 11 fragments. Values were expressed as the mean ± standard deviation, and statistical analyses (JMP, Version 5, SAS, Cary NC) implemented the Student’s *t*-test with p<0.05 judged as significant.

## Results

Figure 1 summarizes the significance of proteolysis with trypsin in order to dissociate DNA from the nuclear DNA binding components. When the purified sperm with progressive motility were suspended in the cell lytic reagents containing Cyber-Gold, the sperm heads were swollen within a few minutes, whereas the DNA fibers still remained in the swollen heads (Fig. 1-A). Addition of trypsin (20 µg/mL) allowed DNA fibers to protrude from the head; their unregulated free diffusion in the solution was, however, not optimal to observe the fragments individually (Fig. 1-B). DNA profiles after cell lysis with trypsin and subsequent SCPFGE were compared between the embedded sperm in the purified fraction and the ejaculates (Fig. 2). Almost all the sperm in the purified fraction showed a mass of tangled DNA fibers with a shaggy surface (Fig. 2-A). In contrast, the ejaculates displayed a different profile, with numerous granular DNA fragments being dispersed in the agarose (Fig. 2-B). This was recognized to be the advanced stage of DNA fragmentation. The results of SCPFGE suggested that the sperm in the purified fraction showed uniformly elongated fibers in several dozen quantities from each tangled mass (Fig. 2-C). The ejaculates yielded quite heterogeneous electrophoretic images in addition to the representative profile shown in Fig. 2-C. As the fragmentation advanced, the number of fibers that pulled out from the mass of DNA decreased in number and shortened in length; conversely, the number of granular fragments dramatically increased (Fig. 2-D).

Figure 3 shows the electrophoretic profiles of intact DNA fibers and those in the end stage of fragmentation. High magnification photographs demonstrated the presence of elongated DNA fibers near the origin (Fig. 3-A) and the apical region (Fig. 3-B). The pulsed-field impression moved the fibers in a serpentine curve within the agarose. Almost all the DNA had eventually been degraded to granular fragments and had been dispersed concentrically through the porous structure of agarose, and, as a result, the mass at the origin was minimized (Fig. 3-C). The corresponding sperm fanned out the granular fragments, which exhibited heterogeneous electrophoretic mobilities (Fig. 3-D).

Although few sperm with progressive motility fell into the advanced stage, (Fig. 2-C), they were either assigned to the group for which only the long chain fibers from the origin were observed (Fig. 3-B) or to the group for which fibrous fragments were observed beyond the anterior end of the elongated fibers. As summarized in Figs. 4-A, -B, -C, the number and size of the fibrous fragments varied among the sperm. It is noteworthy that our novel SCPFGE method was able to detect a single fibrous fragment (Fig. 4-A), and B and C showed 7 and several dozen fragments, respectively. Figures 5 examined the influence of the endonuclease on the electrophoretic profile of DNA fibers. The sperm in the purified fraction showed the long chain fibers after SCPFGE (Fig. 2C), whereas those incubated with DNase I prior to embedding gave the numerous granular fragments. They might be generated as a consequence of enzymatic cleavage of the long chain fibers.

The sperm that were lysed without trypsin produced quite different DNA profiles from those shown in Fig. 2. In this case, SCPFGE could not pull out the fiber from the purified sperm (Fig. 6-A). Compared to the results observed in Fig. 2-D, a portion of the sperm in the ejaculate discharged a smaller amount of granular DNA without the accompanying fibers (Fig. 6-B).

The effects of alkali on the properties of the DNA fibers are summarized in Fig. 7. The lysed sperm corresponding Fig. 2-A were treated with 0.05 mol/L or 0.1 mol/L NaOH prior to SCPFGE. The electrophoretic profiles of trypsin-treated sperm were entirely different from those without trypsin. The DNA fibers of trypsin-treated sperm were disrupted to granular fragments by 0.05 mol/L NaOH (Fig. 7-A). When the amount of NaOH was increased to 0.1 mol/L, the origin shrunk (Fig. 7-B). After SCPFGE, treatment of the elongated DNA fibers (seen in Fig. 2-C) with 0.1 mol/L NaOH showed that the fibers were completely degraded to granules (Fig. 7-C). In contrast, when the sperm lysed without trypsin were treated with 0.1 mol/L NaOH, they discharged smaller amount of granular fragments (Fig 7-D) than those observed in Fig. 7-B.

To determine the number of fragments in the purified sperm with progressive motility, the ejaculates (n = 8, vol. = 2.5±0.62 mL; conc. = 58±26×10^6^ sperm/mL) were processed as described in the Methods, then the resulting fractions (vol. = 1.0 mL; conc. = 5.3±2.7×10^6^ sperm/mL) were analyzed by SCPFGE. As summarized in Table 1, the motility was improved to be more than 90 % in all specimens regardless of their initial values, whereas the percentages of sperm without any fragment were lower significantly (*P*<0.01) than the corresponding values of motility. In the worst case, approximately 40% sperm involved the damaged DNA, despite the motility was found to be 96%. The ratio between the sperm with 1–10 fragments and those with more than 11 fragments were varied widely among the specimens. Table 1 suggested that at least a portion of the motile sperm population had already entered in the early stage of DNA fragmentation.

## Discussion

Pulsed-field gel electrophoresis (PFGE) [20], [21] facilitates the electrophoretic analyses of large DNA molecules through their partial cleavages with restriction endonucleases and through pulsed-field impression with multiple electrode pairs. Our newly developed method of SCPFGE with dual electrode pairs achieved the simultaneous observation of the long-chain fibers, the fibrous and granular fragments derived from a single sperm nucleus. Protamine [22] is an arginine-rich, major nucleoprotein in the mature sperm, DNA-protamine [22] complex is further fixed to the nuclear matrix [23]–[25]. As shown in Fig. 1-A, as little as 8.2 mmol/L hexa-metaphosphate, a polyvalent anion, competitively dissociated the complex, and formed the so-called nuclear halos [26], whereas the DNA fibers were still fixed to the nuclear matrix. Trypsin digestion allowed free diffusion or electrophoretic elongation of the fibers. Proteolysis of the embedded sperm had to be started after gelation of agarose to avoid free diffusion of the fibers (Fig. 1-B). Because the activity of trypsin is strictly dependent on pH, it was kept inactive at pH 4.7 until gelation was completed and was activated by immersing the gel into the cell lytic reagents (pH 8.1).

The overall electrophoretic profiles in Figs. 2–4 elucidated the course of DNA fragmentation. The intact DNA consisted of long chain fibers; at first, several cleavages produced large fibrous fragments. They were shredded to the granular fragments as the fragmentation advanced, finally, the mass at the origin was minimized. The degradation have advanced gradually due to the action of some endogenous nucleases (Fig. 5). In contrast, the comet assay [15], [16] which lysed the sperm without trypsin revealed the electrophoretic behavior of the granular fragments, whereas the fibrous fragments shown in the early stage of DNA fragmentation could not be analyzed by this method (Figs. 1, 2, 4 and 6). An alkali comet assay performed to detect single-strand breaks and alkali-labile sites in the DNA recommended the treatment of the embedded cells with more than 0.1 mol/L NaOH [17], [27]. Our results suggested that alkali treatment chemically cleaved DNA (Fig. 7), and the granular fragments were obvious at 0.05 mol/L NaOH. The sperm without proteolysis (Fig. 7-D) discharged a smaller amount of granular fragments than did those with the treatment (Fig. 7-B), this fact suggested that the nuclear matrix was sensitive to trypsin, but might be resistant to alkali treatment and hence retained a greater part of the DNA at the origin.

Motility has been the most emphasized feature used to assess sperm normality. As summarized in Table 1, SCPFGE revealed that the commonly used procedures for separating motile sperm could exclude the sperm which have already disrupted DNA in the form of granular fragments, and the motile sperm in the prepared fractions may still be contaminated with sperm in the early stage of DNA fragmentation. The conventional ICSI usually depends on intra-operative light microscopic observations to select the appropriate sperm for injection. Our results revealed that sperm motility could not offer complete assurance of DNA integrity, and that molecular biological findings of sperm evaluation should be carefully reviewed. SCPFGE is likely to serve as a fundamental step in the preoperative differential diagnosis to determine the competence of the sperm population provided for injection and is likely to play an important role in ensuring the safety of clinical ICSI.
